# Demographic and Clinical Characteristics of Malignant Solitary Fibrous Tumors: A SEER Database Analysis

**DOI:** 10.3390/cancers16193331

**Published:** 2024-09-29

**Authors:** Mattia Luca Piccinelli, Kyle Law, Reha-Baris Incesu, Stefano Tappero, Cristina Cano Garcia, Francesco Barletta, Simone Morra, Lukas Scheipner, Andrea Baudo, Zhe Tian, Stefano Luzzago, Francesco Alessandro Mistretta, Matteo Ferro, Fred Saad, Shahrokh F. Shariat, Luca Carmignani, Sascha Ahyai, Nicola Longo, Alberto Briganti, Felix K. H. Chun, Carlo Terrone, Derya Tilki, Ottavio de Cobelli, Gennaro Musi, Pierre I. Karakiewicz

**Affiliations:** 1 Cancer Prognostics and Health Outcomes Unit, Division of Urology, University of Montréal Health Center, Montréal, QC H2X 3E4, Canada; 2 Department of Urology, IEO European Institute of Oncology, IRCCS, Via Ripamonti 435, 20141 Milan, Italy; 3 School of Urology, Università degli Studi di Milano, 20122 Milan, Italy; 4 Martini-Klinik Prostate Cancer Center, University Hospital Hamburg-Eppendorf, 20251 Hamburg, Germany; 5 Department of Urology, IRCCS Policlinico San Martino, 16132 Genova, Italy; 6 Department of Surgical and Diagnostic Integrated Sciences (DISC), University of Genova, 16126 Genova, Italy; 7 Department of Urology, University Hospital Frankfurt, Goethe University Frankfurt am Main, 60629 Frankfurt am Main, Germany; 8 Division of Experimental Oncology/Unit of Urology, URI, Urological Research Institute, IRCCS San Raffaele Scientific Institute, 20132 Milan, Italy; 9 Department of Neurosciences, Science of Reproduction and Odontostomatology, University of Naples Federico II, 80131 Naples, Italy; 10 Department of Urology, Medical University of Graz, 8010 Graz, Austria; 11 Department of Urology, IRCCS Policlinico San Donato, 20097 Milan, Italy; 12 Department of Oncology and Haemato-Oncology, Università degli Studi di Milano, 20122 Milan, Italy; 13 Department of Urology, Comprehensive Cancer Center, Medical University of Vienna, 1090 Vienna, Austria; 14 Department of Urology, Weill Cornell Medical College, New York, NY 10021, USA; 15 Department of Urology, University of Texas Southwestern Medical Center, Dallas, TX 75390, USA; 16 Hourani Center of Applied Scientific Research, Al-Ahliyya Amman University, Amman P.O. Box 19328, Jordan; 17 Department of Urology, Istituto di Ricovero e Cura a Carattere Scientifico Ospedale Galeazzi—Sant’Ambrogio, 20157 Milan, Italy; 18 Department of Urology, University Hospital Hamburg-Eppendorf, 20251 Hamburg, Germany; 19 Department of Urology, Koc University Hospital, 20251 Istanbul, Turkey

**Keywords:** solitary fibrous tumor, competing risks analyses, tumor-size cut-offs

## Abstract

**Simple Summary:**

Solitary fibrous tumors represent a rare mesenchymal malignancy that can occur anywhere in the body. Due to the low prevalence of the disease, there is a lack of contemporary data regarding patient demographics and cancer-control outcomes. We validated the importance of stage and surgical resection as independent predictors of cancer-specific mortality in malignant solitary fibrous tumors. Moreover, we provide novel observations regarding the independent importance of tumor size, regardless of the site of origin, stage and/or surgical resection status.

**Abstract:**

Background/Objectives: Solitary fibrous tumors (SFTs) represent a rare mesenchymal malignancy that can occur anywhere in the body. Due to the low prevalence of the disease, there is a lack of contemporary data regarding patient demographics and cancer-control outcomes. Methods: Within the SEER database (2000–2019), we identified 1134 patients diagnosed with malignant SFTs. The distributions of patient demographics and tumor characteristics were tabulated. Cumulative incidence plots and competing risks analyses were used to estimate cancer-specific mortality (CSM) after adjustment for other-cause mortality. Results: Of 1134 SFT patients, 87% underwent surgical resection. Most of the tumors were in the chest (28%), central nervous system (22%), head and neck (11%), pelvis (11%), extremities (10%), abdomen (10%) and retroperitoneum (6%), in that order. Stage was distributed as follows: localized (42%) vs. locally advanced (35%) vs. metastatic (13%). In multivariable competing risks models, independent predictors of higher CSM were stage (locally advanced HR: 1.6; metastatic HR: 2.9), non-surgical management (HR: 3.6) and tumor size (9–15.9 cm HR: 1.6; ≥16 cm HR: 1.9). Conclusions: We validated the importance of stage and surgical resection as independent predictors of CSM in malignant SFTs. Moreover, we provide novel observations regarding the independent importance of tumor size, regardless of the site of origin, stage and/or surgical resection status.

## 1. Introduction

Solitary fibrous tumors (SFTs) represent a rare mesenchymal malignancy that accounts for <2% of all soft-tissue sarcomas and can occur anywhere in the body [1,2,3]. Cellular tumors, which were formerly known as hemangiopericytomas, are now considered to be part of the SFT spectrum [2,4,5,6,7,8]. Although indicators of more aggressive treated natural history consist of elevated mitotic index, infiltrative margins, hypercellularity, pleomorphism and necrosis (specifically, a proposed definition of malignant SFTs is tumors with focal areas of marked increased cellularity described as greater than 5% of tumor that are devoid of alternating sclerotic areas and have greater than four mitoses per ten high-powered fields [1]), no consensus exists regarding the treated natural history when such features are absent from pre-treatment biopsy specimens. In consequence, the search for accurate and reliable predictors of treated natural history in SFTs is ongoing [2,3,4,9,10]. In that regard, only small case series (*n* = 110–219) have been published, and these suggest a 4–19% rate of 10-year local recurrence and a 13–45% rate of 10-year metastatic progression [6,11,12]. Similarly, survival patterns according to the site of origin, stage and surgical resection status are also based on very limited data [6,12,13,14,15,16,17]. Last but not least, no systematic assessment of tumor-size cut-offs for the prediction of cancer-specific mortality (CSM) has ever been performed to date. We addressed these knowledge gaps, relying on the 2000–2019 Surveillance, Epidemiology, and End Results (SEER) database [18]. We tested whether stage, surgical resection and possibly tumor size represent predictors of CSM across different sites of origin of primary malignant SFTs. Moreover, we hypothesized that significant differences in patient characteristics and CSM rates exist according to the site of origin, stage, surgical resection status and tumor size.

## 2. Materials and Methods

### 2.1. Patient Characteristics

Within the SEER database (2000–2019), we focused on patients aged ≥18 who harbored malignant SFTs (International Classification of Disease for Oncology histology code 8815/3 and 9150/3 [4,7]) and had known follow-up and primary site. SFT origin was tabulated according to SEER location (central nervous system, extremities, head and neck, chest, pelvis, abdomen and retroperitoneum [6,11,12,13,14,17,19]) and SEER stage (localized, locally advanced and metastatic [14,15,17,18,20]). Specifically, SEER staging defines localized cancer as that limited to the organ in which it began, without evidence of spread. SEER staging defines locally advanced (or regional) cancer as that which has spread beyond the primary site to nearby lymph nodes or organs and tissues. Metastatic (or distant) cancer is defined as a disease that has spread from the primary site to distant organs or distant lymph nodes. Tumor size was stratified as follows: <9 cm, 9–15.9 cm and ≥16 cm.

### 2.2. Statistical Analysis

Descriptive statistics were used to characterize patient age and tumor size and stage. Cumulative incidence plots depicted CSM rates after adjustment for other-cause mortality (OCM). Moreover, we tested for the ideal tumor-size cut-off for the prediction of CSM using a minimum *p*-value approach. Subsequently, univariable and multivariable competing risks regression models were used to test for independent predictors of CSM after adjustment for OCM. All statistical tests were two-sided, with the level of significance set at *p* < 0.05, and were performed with R Software Environment for Statistical Computing and Graphics (R version 4.1.3, R Foundation for Statical Computing, Vienna Austria) [21].

## 3. Results

### 3.1. Patient and Tumor Characteristics in the Overall Cohort

Of 1134 patients with malignant SFTs, 551 (49%) were male and 989 (87%) were surgically treated (Table 1).

**Figure 1:**
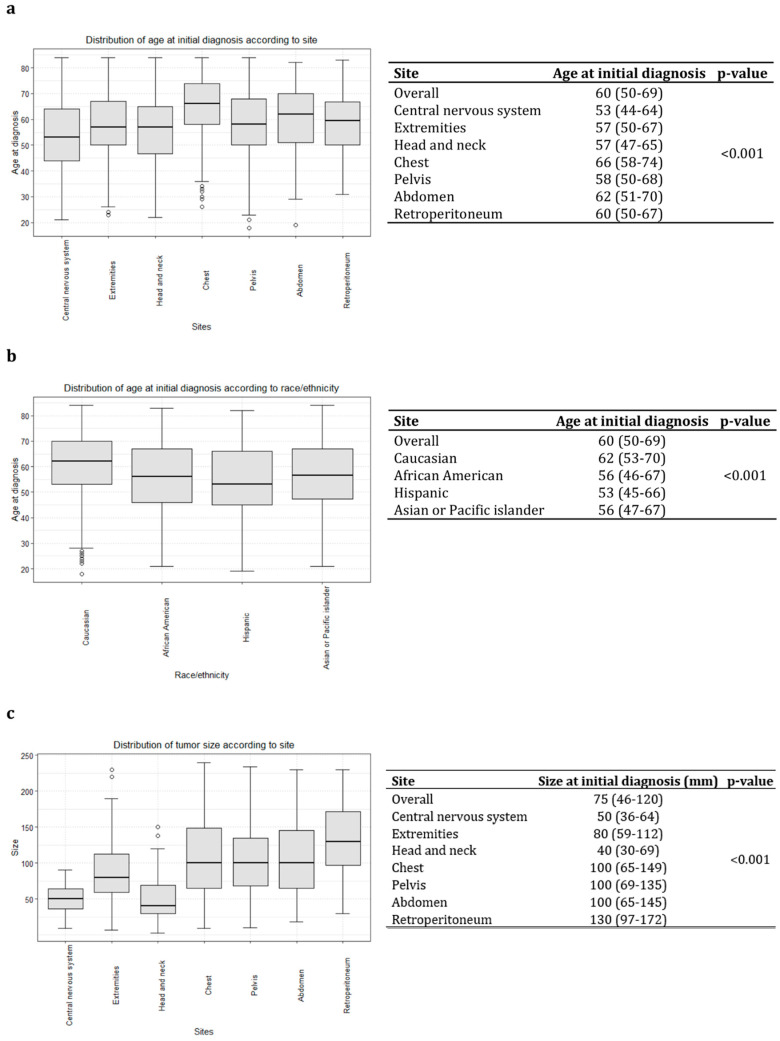
Whisker plots depicting the distribution of patients diagnosed with malignant solitary fibrous tumors, as recorded in the 2000–2019 Surveillance, Epidemiology, and End Results database: (**a**) age at diagnosis according to tumor site of origin; (**b**) age at diagnosis according to race/ethnicity; (**c**) tumor size (mm) according to tumor site of origin.

The median age at diagnosis was 60 years (Figure 1a).

Most SFTs were located in the chest (*n* = 322, 29%), central nervous system (*n* = 261, 23%), head and neck (*n* = 120, 11%), pelvis (*n* = 128, 11%), extremities (*n* = 119, 10%), abdomen (*n* = 114, 10%) and retroperitoneum (*n* = 70, 6%), in that order (Supplementary Table S1). Overall, 475 (42%) of patients harbored localized tumors, while 401 (35%) had tumors that were locally advanced and 142 (13%) had tumors in the metastatic stage. In 116 (10%) patients, the stage was unknown. Median tumor size was 75 mm (IQR: 46–120).

### 3.2. Patient and Tumor Characteristics According to the Site of Origin

**Figure 2:**
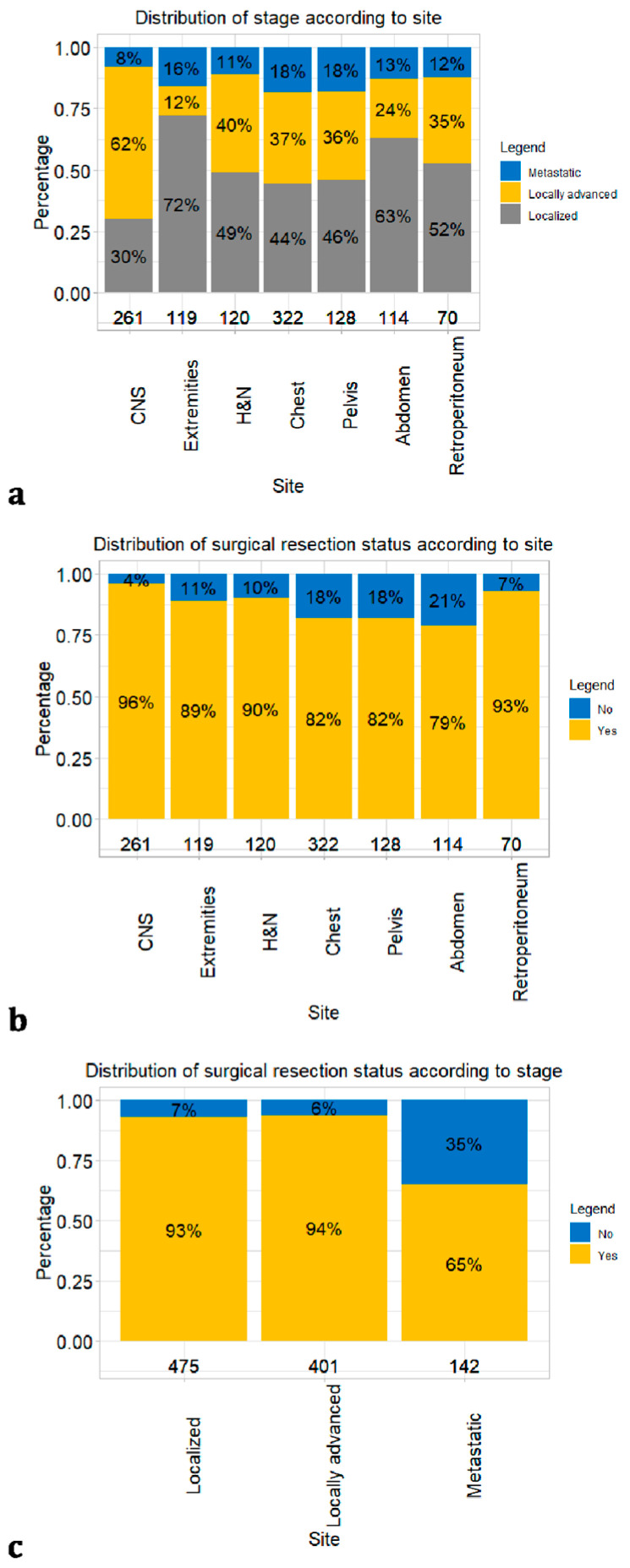
Bar plot depicting the distribution of patients diagnosed with malignant solitary fibrous tumors, as recorded in the 2000–2019 Surveillance, Epidemiology, and End Results database: (**a**) stage according to site; (**b**) surgical resection according to site; (**c**) surgical resection according to stage.

Differences in stage distribution were recorded according to the site of origin. SFTs in the extremities were the most frequently localized (*n* = 77, 72%, Figure 2a).

Conversely, SFTs in the central nervous system were the most frequently locally advanced (*n* = 152, 62%). The frequency of metastatic SFTs ranged from 8 (*n* = 20, central nervous system) to 18% (*n* = 51 in the chest and *n* = 21 in the pelvis). Rates of surgical resection status also varied according to the site of origin (Figure 2b). Specifically, 251 (96%) SFTs were surgically resected from the central nervous system, which was followed in frequency of resection by the retroperitoneum (*n* = 65, 93%), head and neck (*n* = 108, 90%), extremities (*n* = 106, 89%), chest (*n* = 264, 82%), pelvis (*n* = 105, 82%) and abdomen (*n* = 90, 79%), in that order. Finally, according to stage, 444 (93%) patients with localized SFTs vs. 375 (94%) patients with locally advanced SFTs vs. 92 (65%) patients with metastatic SFTs underwent surgical resection (Figure 2c). In general, the smaller SFTs had the head and neck (median size: 40 mm) and central nervous system (median size: 50 mm) as sites of origin. Conversely, the larger tumors had the chest (median size: 100 mm), pelvis (median size: 100 mm) and abdomen (median size: 100 mm) as sites of origin. Finally, the largest SFTs originated in the retroperitoneum (median size: 130 mm, Figure 1c).

### 3.3. Cancer-Specific and Other-Cause Mortality in Solitary Fibrous Tumor

**Figure 3:**
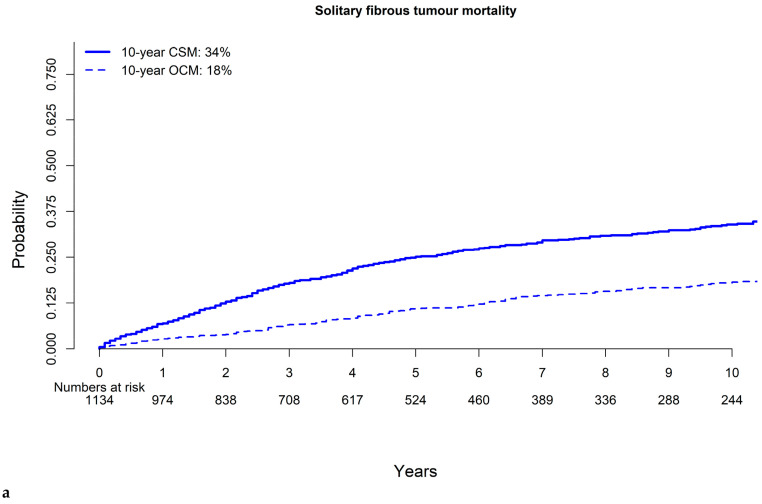

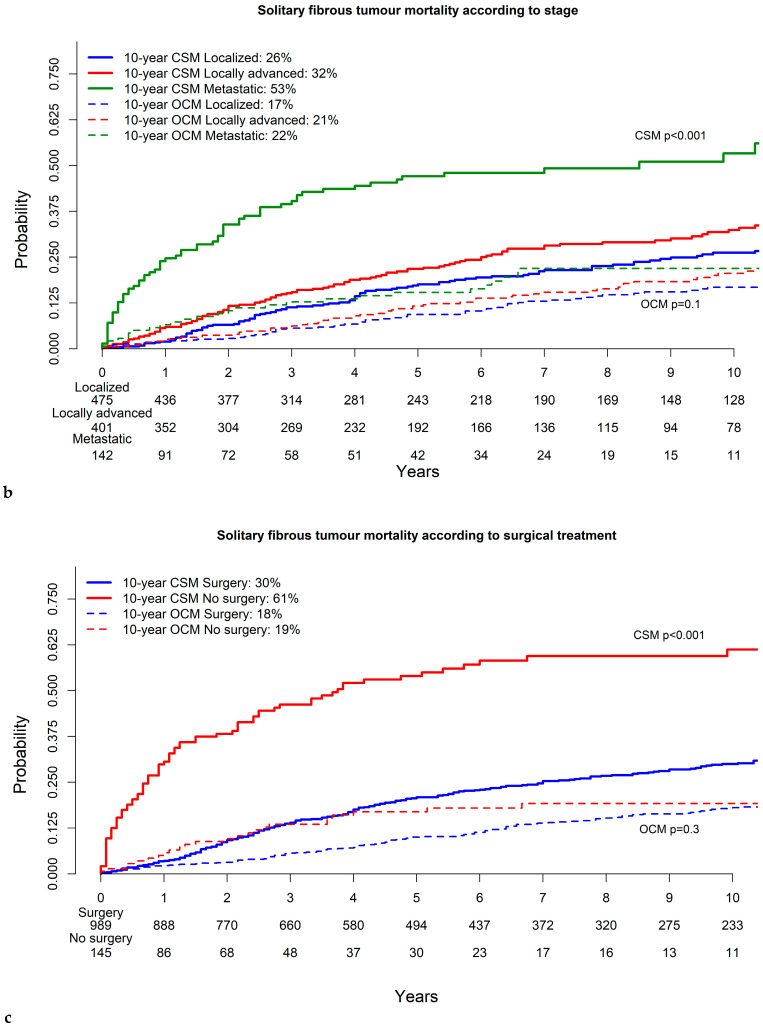

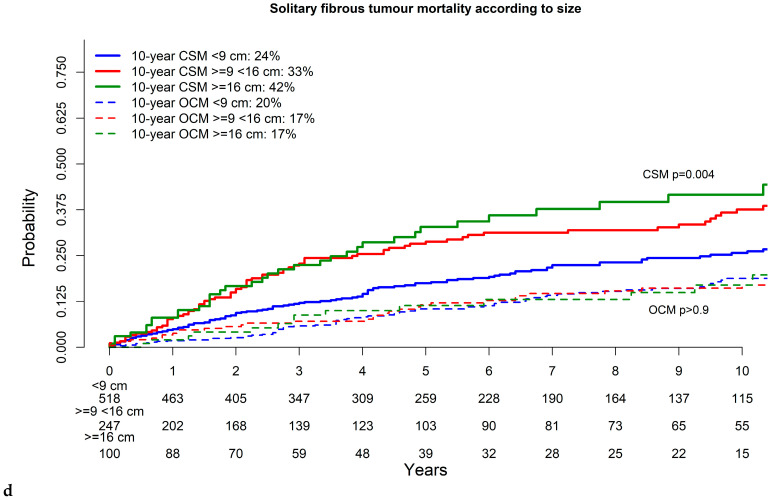
Cumulative incidence plots depicting cancer-specific mortality and other-cause mortality over 10 years in patients diagnosed with malignant solitary fibrous tumors in 2000–2019 according to the Surveillance, Epidemiology, and End Results database (**a**) overall and according to (**b**) stage; (**c**) surgical resection status; (**d**) tumor size.

In cumulative incidence plots, based on 1134 malignant SFT patients, 10-year CSM and OCM rates were 34 and 18%, respectively (Figure 3a).

Specifically, the lowest 10-year CSM rate was recorded for SFTs located in the head and neck (21%), with the second-lowest rate being that for SFTs located in the central nervous system (26%). Conversely, the highest rate of 10-year CSM was recorded for SFTs located in the abdomen (44%, Supplementary Figure S1a). CSM rates at ten years according to stage were 26% in localized vs. 32% in locally advanced vs. 53% in metastatic SFTs (Figure 3b). Ten-year CSM rates according to surgical resection status (yes vs. no) were 30 vs. 61%, respectively (Figure 3c). Finally, 10-year CSM rates according to tumor-size intervals were as follows: tumor size <9 cm 24%; tumor size 9–15.9 33%; tumor size ≥16 cm 42% (Figure 3d). In multivariable competing risks analyses, locally advanced stage (hazard ratio [HR]: 1.6, *p* < 0.001), metastatic stage (HR: 2.9, *p* < 0.001), non-surgical management (HR: 3.6, *p* < 0.001) and tumor size (9–15.9 cm HR: 1.6, *p* = 0.01; ≥16 cm HR: 1.9, *p* = 0.01) independently predicted higher CSM rates after additional adjustment for OCM (Table 2).

Conversely, after adjustment for age at diagnosis, stage, surgical resection status and tumor size and additional adjustment for OCM, the site of origin failed to achieve independent predictor status for CSM. Finally, in separate multivariable competing risks analyses testing surgical resection status according to stage, non-surgical management achieved independent predictor status for higher CSM in localized (HR: 1.8, *p* = 0.03), locally advanced (HR: 2.6, *p* = 0.01) and metastatic (HR: 5.1, *p* < 0.001) SFTs (Table 3).

## 4. Discussion

Small series studies have explored SFT survival patterns according to the site of origin, stage, surgical resection status and tumor size [6,12,13,14,15,16,17]. However, no comprehensive and systematic assessment of tumor-size cut-offs for the prediction of CSM has ever been reported. We addressed these knowledge gaps and hypothesized that significant differences in patient characteristics and CSM rates exist according to the site of origin, stage, surgical resection status and tumor size. Several important observations were made.

First, we provided the most detailed tabulation of sites of origin within the largest (*n* = 1134) and most contemporary (year of diagnosis: 2000–2019) cohort of patients with malignant SFTs. We identified the chest (28%) as the most frequent site of origin, followed by the central nervous system (22%). The rates of SFT origin were virtually equally distributed between head and neck (11%), pelvis (11%), extremities (10%) and abdomen (10%). Conversely, retroperitoneal SFTs were the least frequent (6%). Based on the absence of previously published detailed data regarding sites of SFT origin, our observations can only be partially compared to other smaller and more historical series [6,11,12,13,14,16,17,19,22,23,24]. However, these comparisons revealed a close agreement between the current rates and historical rates from smaller series.

The current database also allowed us to tabulate SFTs according to the stage and provided the most robust and contemporary results. Specifically, of all SFTs, 42% were localized, 35% were locally advanced and 13% were metastatic. Unfortunately, we also observed that 10% were of unknown stage. The percentage of SFTs of unknown stage is comparable to percentages of tumors of unknown stage in the SEER database for other malignancies such as kidney [25] or prostate [26] cancers. The distribution of SFT patients across stages differed from that found in the study of Wang et al. [15] (*n* = 1243, year of diagnosis: 1975–2016), which relied on a more historical SEER cohort. For example, in the analyses by Wang et al., only 17% of patients harbored tumors in the locally advanced stage vs. 35% in the current cohort. This discrepancy may be explained by a very elevated rate of tumors of unknown stage in the Wang et al. cohort: 35%, vs. 10% in the current study.

Finally, we provided the most generalizable distribution of surgical resection status. Overall, 87% of patients had undergone surgery. This observation is in perfect agreement with that in the historical cohort of Wang et al. [15], where surgical resection was accomplished in 88% of SFT patients. However, in the Wang et al. study, surgical resection status was not stratified according to the SFT site of origin and stage. We addressed this knowledge gap in the current study. Specifically, the highest rate of surgical resection was recorded for tumors in the central nervous system (96%), followed by those in the retroperitoneum (93%), head and neck (90%), extremities (89%), chest (82%), pelvis (82%) and abdomen (79%), in that order. We also provided rates of surgical resection status according to stage. Specifically, 444/475 (93%) patients with localized SFTs underwent surgical resection vs. 375/401 (94%) patients with locally advanced SFTs and 92/142 (65%) patients with metastatic SFTs. The very elevated surgical resection rates associated with localized and locally advanced SFTs (93–94%) validate the pivotal role of surgery. Additionally, the central role of surgery was also confirmed in patients with metastatic SFTs, the vast majority (65%) of whom underwent resection.

Second, we are the first to validate that the absence of surgical resection independently predicts higher CSM (HR: 3.6, *p* < 0.001) in malignant SFTs of all stages after adjustment for OCM. Moreover, in stage-specific analyses, absence of surgical resection also independently predicted higher CSM. Specifically, for localized SFTs, absence of surgical resection exhibited an HR of 1.8 (*p* = 0.03); this value was 2.6 (*p* = 0.01) in the locally advanced stage and 5.1 (*p* < 0.001) in the metastatic stage. These observations validate the central role of surgical resection in the contemporary management of SFTs at all stages. Moreover, the increase in the magnitude of HRs from the localized to the locally advanced to the metastatic stages adds further evidence supporting the disadvantage of non-surgical management, which is most pronounced in metastatic patients.

Third, we also provided the most contemporary and generalizable validation of the importance of stage as a predictor of CSM. Here, relative to the localized stage, patients with locally advanced SFTs exhibited an HR of 1.6 (*p* < 0.001); this value was 2.9 (*p* < 0.001) in the metastatic stage. Unfortunately, direct comparisons with other series regarding the effect of the stage, as well as of the effect of surgical resection status according to specific SFT stage, cannot be made. For example, Wushou et al. [14] addressed only hemangiopericytoma, which represents only a subset of currently diagnosed malignant SFTs. Moreover, no previous studies relied on competing risks analyses adjusting for OCM when the stage was tested in a multivariable fashion.

Fourth, we are the first to perform a comprehensive and systematic assessment of tumor-size cut-offs for the prediction of CSM in malignant SFTs. Here, tumor-size cut-offs of <9, 9–15.9 and ≥16 cm emerged as ideal. Moreover, their independent predictor status was confirmed in multivariable analyses that included age at diagnosis, site of origin, stage, surgical resection status and additional adjustment for OCM. Our findings should ideally be validated within an equally large or even larger population-based data repository. Previous analyses regarding tumor size relied on the median [11,12,16,27] or generally accepted tumor-size cut-offs [28,29,30] used for other primary tumors, such as retroperitoneal sarcoma [31]. However, none of these previous smaller-scale (*n* = 110–239) analyses questioned the internal validity of such definitions for SFT patients. The most widely used and established cut-offs were defined by Demicco et al. [6] and are included in their scoring system to predict distant metastasis. Specifically, this innovative and elegant study provided the basis for the contemporary management of SFTs. The authors (Demicco et al.) relied on 110 SFT patients treated at M.D. Anderson Cancer Center (1986–2009). For analysis purposes, tumor-size cut-offs of <5, 5–9.9, 10–14.9 and ≥15 cm were used. However, these cut-off values were not based on specific clinical or statistical criteria. Instead, they may have been adopted from values used for other primary tumors such as retroperitoneal sarcoma [31]. Finally, the independent predictor status for these tumor-size cut-offs (<5, 5–9.9, 10–14.9 and ≥15 cm) was not tested regarding CSM. Similar methodological limitations regarding testing of tumor-size cut-offs also apply to the study by Gholami et al. [12]. Here, the authors relied on a single tumor-size cut-off of 8 cm within a historical cohort (1982–2015) of 219 SFT patients treated at Memorial Sloan Kettering Cancer Center. Importantly, the majority (74%) of the patients in the Gholami et al. study harbored non-malignant SFTs. In consequence, the proposed tumor-size cut-off is predominantly applicable to non-malignant SFTs, not to malignant SFTs.

Fifth, we tested for OCM rates and, although malignant SFT is associated with high rates of 10-year CSM, some patients died of other causes. Based on the absence of data quantifying OCM rates in SFTs, we provide values numbers as follows: 5-year OCM 11% and 10-year OCM 18%. Since OCM affects a non-negligible portion of SFT patients, ideally, competing risks analyses should be preferred when CSM rates are addressed.

Overall, the present study is based on the largest and most contemporary malignant SFT cohort analyzed to date and provides the most robust, comprehensive and detailed analyses of patient- and tumor-associated risk factors that may affect CSM. Three variables emerged as independent predictors of CSM in multivariable competing risks models that also adjusted for OCM: stage, surgical resection status and tumor size. We relied on a minimum *p*-value approach to explore potential tumor-size cut-offs. Ideal tumor-size cut-offs of <9, 9–15.9 and ≥15 emerged and represented independent predictors of CSM. However, these tumor-size cut-offs ideally require independent validation within an external cohort.

Despite its novelty, our study is not devoid of limitations. First, the SEER is a retrospective database with the potential for selection biases. However, observational databases such as SEER or NCDB represent the only opportunity to study rare primary tumors and reach statistically robust conclusions. Second, rates of local recurrence, metastatic progression and preoperative and postoperative treatments, as well as predictors of cancer-control outcomes (such as mitotic count, tumor necrosis and positive surgical margins) are not available in the SEER database. In consequence, our results should be tested and validated in other large-scale cohorts of patients with malignant SFTs. Fourth, SEER lacks specific baseline comorbidity information. In consequence, more detailed analyses adjusting for comorbidities were not possible. However, we partially addressed this limitation by the inclusion of OCM rates in our analyses.

## 5. Conclusions

We validated the importance of stage and surgical resection as independent predictors of cancer-specific mortality in malignant solitary fibrous tumors. Moreover, we provided novel observations regarding the independent importance of tumor size, regardless of the site of origin, stage and/or surgical resection status.

## Figures and Tables

**Table 1:** Descriptive characteristics of patients diagnosed with malignant solitary fibrous tumors between 2000 and 2019, as recorded in the Surveillance, Epidemiology, and End Results database. Data are shown as medians for continuous variables or as counts and percentages (%) for categorical variables. IQR: interquartile range.

Malignant Solitary Fibrous Tumor	Overall *n* = 1134
Age at diagnosis (years) Median (IQR)	60 (50–69)
Sex—Male	551 (49%)
Race/ethnicity	
Caucasian	771 (68%)
African American	95 (8%)
Hispanic	149 (13%)
Asian/Pacific Islander	102 (9%)
Other	17 (2%)
Surgical resection	989 (87%)
Site of origin	
Extremities and head	500 (44%)
Central nervous system	261 (23%)
Head and neck	120 (11%)
Extremities	119 (10%)
Chest	322 (29%)
Infradiaphragmatic	312 (28%)
Pelvis	128 (11%)
Abdomen	114 (10%)
Retroperitoneum	70 (6%)
Size (cm)	
Median (IQR)	75 (46–120)
<9 cm	518 (46%)
9–15.9 cm	247 (22%)
≥16 cm	100 (9%)
Unknown	269 (24%)
Stage	
Localized	475 (42%)
Locally advanced	401 (35%)
Metastatic	142 (13%)
Unstaged	116 (10%)

**Table 2:** Univariable and multivariable competing risks analyses predicting cancer-specific mortality and accounting for other-cause mortality. All patients were diagnosed with malignant solitary fibrous tumors between 2000 and 2019, as recorded in the Surveillance, Epidemiology, and End Results database.

	Univariable			Multivariable		
Variables Tested	Hazard Ratio	95% CI	*p*-Value	Hazard Ratio	95% CI	*p*-Value
Age at diagnosis (years)	1.01	(1.01–1.02)	<0.001	1.01	(1–1.02)	0.08
Sex—Female	0.92	(0.7–1.2)	0.5			
Race/ethnicity						
Caucasian	Ref					
African American	1.2	(0.7–1.9)	0.5			
Hispanic	0.7	(0.4–1.1)	0.1			
Asian or Pacific Islander	0.9	(0.6–1.4)	0.7			
Surgical resection status—No	4	(2.7–6.1)	<0.001	3.6	(2.3–5.6)	<0.001
Site of origin						
Central nervous system	Ref			Ref		
Extremities	1.6	(0.98–2.7)	0.06	1.6	(0.9–2.9)	0.1
Head and neck	1.3	(0.7–2.2)	0.4	1.2	(0.7–2.1)	0.6
Chest	1.7	(1.1–2.5)	0.01	0.97	(0.6–1.7)	0.9
Pelvis	1.5	(0.9–2.5)	0.11	0.9	(0.5–1.7)	0.8
Abdomen	1.7	(0.99–3)	0.06	1.3	(0.7–2.5)	0.5
Retroperitoneum	1.3	(0.6–2.5)	0.52	0.8	(0.3–1.6)	0.5
Size						
<9 cm	Ref			Ref		
9–15.9 cm	1.5	(1.1–2.0)	0.01	1.6	(1.1–2.4)	0.01
≥16 cm	1.8	(1.2–2.6)	<0.001	1.9	(1.1–3.1)	0.01
Stage						
Localized	Ref			Ref		
Locally advanced	1.5	(1.1–2)	0.02	1.6	(1.2–2.3)	<0.001
Metastatic	3.4	(2.3–5)	<0.001	2.9	(2.0–4.4)	<0.001

**Table 3:** Separate multivariable competing risks analyses testing the independent CSM predictor status of surgical resection status after adjustment for OCM according to stage. All models were adjusted for age at diagnosis, site of origin and tumor size. All patients were diagnosed with malignant solitary fibrous tumors between 2000 and 2019, as recorded in the Surveillance, Epidemiology, and End Results database.

	Localized			Locally Advanced			Metastatic		
Variables Tested	Hazard Ratio	95% CI	*p*-Value	Hazard Ratio	95% CI	*p*-Value	Hazard Ratio	95% CI	*p*-Value
Surgical resection status—No	1.8	(0.6–5.1)	0.03	2.6	(1.3–5.3)	0.01	5.1	(2.6–9.8)	<0.001

## Data Availability

Data available in a publicly accessible repository.

## Supplementary Materials

The following supporting information can be downloaded at: https://www.mdpi.com/article/10.3390/cancers16193331/s1. Figure S1: Cumulative incidence plots and summary table depicting cancer-specific mortality and other-cause mortality over 10 years in patients with malignant solitary fibrous tumors recorded in the 2000–2019 Surveillance, Epidemiology, and End Results database according to (a) site, (b) location. Table S1: Distribution of organ of origin among 198 patients diagnosed with pelvis and retroperitoneum solitary fibrous tumor between 2000 and 2019, as recorded in the Surveillance, Epidemiology, and End Results database.
