# Supplementary material for: Demographic and Clinical Characteristics of Malignant Solitary Fibrous Tumors: A SEER Database Analysis
Source: Cancers (Basel). 2024 Sep 29;16(19):3331. doi: 10.3390/cancers16193331 (PMC11482613; doi:10.3390/cancers16193331)
Supplement: Supplementary file 1 [file cancers-16-03331-s001.zip › cancers-3155638-supplementary.pdf]

Site	5-year CSM	10-year CSM	5-year OCM	10-year OCM
**Overall**	25%	34%	11%	18%
**Central nervous system**	16%	26%	10%	21%
**Extremities**	26%	38%	7%	8%
**Head and neck**	17%	21%	7%	16%
**Chest**	32%	39%	14%	23%
**Pelvis**	26%	39%	12%	15%
**Abdomen**	35%	44%	13%	17%
**Retroperitoneum**	22%	30%	10%	16%

a

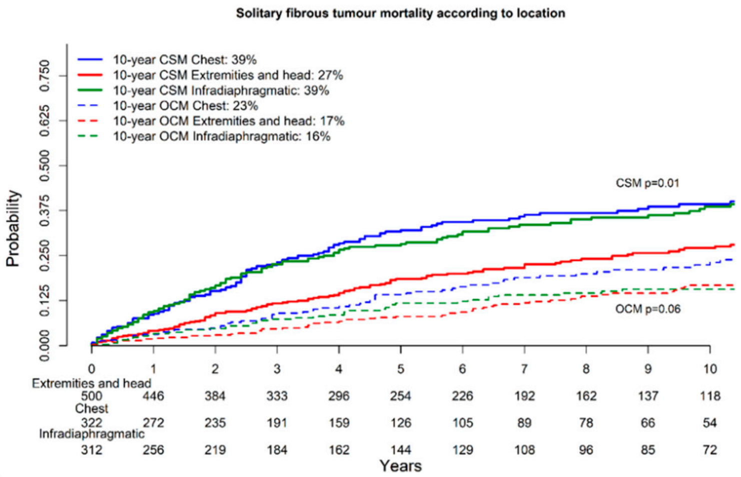

b

Supplementary Figure S1. Cumulative incidence plots and summary table depicting cancer-specific mortality and other-cause mortality over 10 years in patients with malignant solitary fibrous tumor diagnosed in 2000-2019 Surveillance, Epidemiology, and End Results database according to: (a) Site; (b) Location.

Supplementary Table S1. Organ of origin distribution in 198 patients diagnosed with pelvis and retroperitoneum solitary fibrous tumor between 2000 and 2019 in the Surveillance, Epidemiology, and End Results database.

	Sites	N (%)
**Pelvis**	Other soft tissues of the pelvis	102 (52%)
	Bones of the pelvis	7 (4%)
	Penis and vulva	8 (4%)
	Prostate	3 (2%)
	Bladder	2 (1%)
	Vagina	2 (1%)
	Ovary	2 (1%)
	Myometrium	1 (1%)
	Testis	1 (1%)
**Retroperitoneum**	Other soft tissues of retroperitoneum	55 (28%)
	Kidney	13 (7%)
	Renal pelvis	1 (1%)
	Adrenal gland	1 (1%)
